# Editorial: Potentials of Kampo Medicine in Modern Society

**DOI:** 10.3389/fnut.2022.912874

**Published:** 2022-06-14

**Authors:** Hajime Nakae, Shin Takayama, Takao Namiki

**Affiliations:** ^1^Department of Emergency and Critical Care Medicine, Akita University Graduate School of Medicine, Akita, Japan; ^2^Department of Education and Support for Regional Medicine, Tohoku University Hospital, Sendai, Japan; ^3^Department of Kampo Medicine, Tohoku University Hospital, Sendai, Japan; ^4^Department of Japanese-Oriental (Kampo) Medicine, Graduate School of Medicine, Chiba University, Chiba, Japan

**Keywords:** traditional Japanese medicine, Kampo, syakuyakukanzoto, keishibukuryogan, hochuekkito

Public education of Traditional Japanese (Kampo) Medicine in Japan was abruptly stopped in 1895. As a result, Kampo went into steep decline. Nevertheless, Kampo medicine has gradually reemerged and now occupies a considerable position in the field of medical practice and education (1, 2). Its unforeseen significant effect is occasionally observed in patients with intractable diseases that western-style medicines don't work at all (3). Moreover, the Kampo concept of tonifying is used in persons with frailty or intractable infections such as *MRSA* or multi-drug-resistant *Pseudomonas aeruginosa* (4, 5).

It is also applicable in both emergency and intensive care units (6). For example, shakuyakukanzoto enables the rapid control of myalgia and is used to treat tetanus-induced convulsions (7). Goreisan is used for fluid^TM1^ disturbance and for the treatment of vertigo and acute gastroenteritis (8). Furthermore, blood ^TM1^ disturbance is effective for the acute treatment of trauma. Hematoma is considered a form of static blood ^TM1^; therefore, formulations that are useful for treating static blood ^TM1^, such as keishibukuryogan and jidabokuippo, can be used (9–11). Satoh and Nakae reported that the administration of daijokito (DJT), which is composed of Magnolia bark, immature orange, rhubarb rhizome, and anhydrous mirabilitum, caused defecation in critically ill patients and significantly increased the stool volume. The anhydrous mirabilitum in DJT has a stool softening effect, and rhubarb rhizome has a hypermotility effect, and they are traditionally utilized couplings. Furthermore, Magnolia bark has psychotropic effects, and immature orange has anti-inflammatory effects. Such synergistic effects and multifunctionality are the strong points of Kampo medicine. While the negative effects of polypharmacy may occur to cover various effects with western medicines, the combination of crude drugs in Kampo medicine has been sophisticated throughout history.

In a medical environment that favors modern Western medicine, treatment with Kampo medicine is not common in emergency and critical care medicine. Nevertheless, treatments for acute infection, poisoning, or resuscitation are described in Shanghan Lun and Jin Gui Yao Lue, regarded as “emergency manuals,” both written by Zhang Zhongjing (150–219). We should apply such manuals as a gift of wisdom from ancestors and use them as suitable for our modern society. Kampo medicine might be applied to coronavirus disease 2019 (COVID-19) as well, since it has been used for viral infections such as influenza (12). Heat-clearing formula such as saikatsugekito is expected to prevent serious illness in mild cases (13–17). Tonic formula such as hochuekkito may prevent infections since it has multiple effects through the digestive and immune systems, including for acute viral infection and chronic inflammation (18, 19).

Thus, the quality of acute and chronic treatment strategies may be improved by taking advantage of all available medical resources and practices such as Western and Kampo medicines (Figure 1).

**Figure 1:**
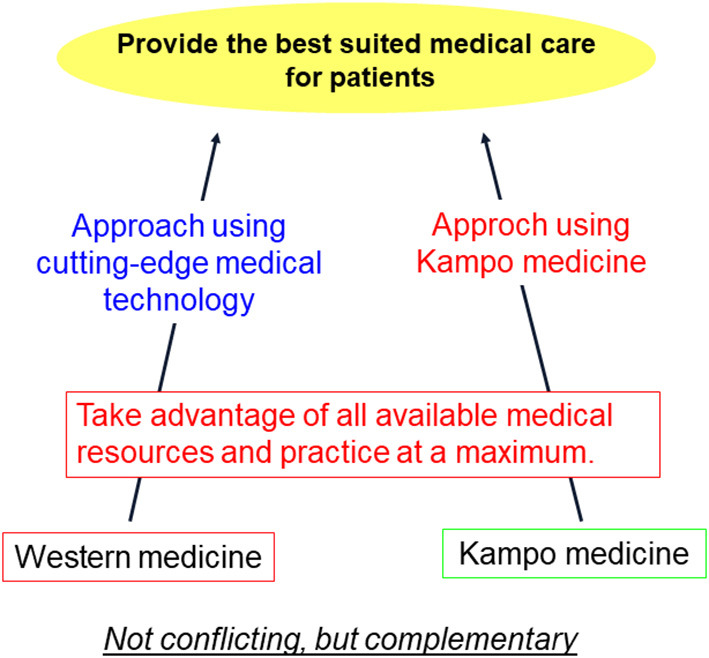
Hybrid-type treatment using Western and Kampo medicine.

Some adverse events that may present a risk of occurring in patients based on the known actions of the major active components of certain drugs are as follows; Ephedra herb, Glycyrrhiza root, Aconite tuber, rhubarb rhizome, and anhydrous mirabilitum (20). Yoshino et al. summarized clinical risk factors of Licorice-induced pseudoaldosteronism in this topic.

Now is the time to recognize Kampo medicine is effective in a variety of medical areas in a modern society.

## Author Contributions

All authors listed have made a substantial, direct, and intellectual contribution to the work and approved it for publication.

## Conflict of Interest

ST belongs to the Department of Kampo and Integrative Medicine, Tohoku University Graduate School of Medicine, a joint research course with TSUMURA & Co. ST and TN received research funding from TSUMURA & Co. The remaining author declares that the research was conducted in the absence of any commercial or financial relationships that could be construed as a potential conflict of interest.

## Publisher's Note

All claims expressed in this article are solely those of the authors and do not necessarily represent those of their affiliated organizations, or those of the publisher, the editors and the reviewers. Any product that may be evaluated in this article, or claim that may be made by its manufacturer, is not guaranteed or endorsed by the publisher.
